# Activity of novel ceftibuten-avibactam, ceftazidime-avibactam, and comparators against a challenge set of *Enterobacterales* from outpatient centers and nursing homes across the United States (2022–2024)

**DOI:** 10.1128/aac.01867-24

**Published:** 2025-04-03

**Authors:** Aliaa Fouad, April M. Bobenchik, Robin Chamberland, Andrew E. Clark, Holly K. Huse, Isabella W. Martin, A. Brian Mochon, Erik Munson, Maroun M. Sfeir, Monica Srodon, Kimberly Taylor, Yungchou Wang, Lars F. Westblade, David P. Nicolau, Tomefa E. Asempa

**Affiliations:** 1 Center for Anti-Infective Research and Development, Hartford Hospital 23893, Hartford, Connecticut, USA; 2 Department of Pathology, Division of Clinical Pathology, Penn State Health Milton S. Hershey Medical Center 12311, Hershey, Pennsylvania, USA; 3 Department of Pathology, Saint Louis University 167138, St. Louis, Missouri, USA; 4 Department of Pathology, The University of Texas Southwestern Medical Center 465773, Dallas, Texas, USA; 5 Department of Pathology, Harbor-UCLA Medical Center 21640, Torrance, California, USA; 6 Department of Pathology and Laboratory Medicine, Dartmouth Health 4456, Lebanon, New Hampshire, USA; 7 Banner Health 3605, Phoenix, Arizona, USA; 8 Sonora Quest Laboratories 273991 , Phoenix, Arizona, USA; 9 Department of Pathology, University of Arizona College of Medicine, Phoenix, Arizona, USA; 10 Department of Medical Laboratory Science, Marquette University 5505, Milwaukee, Wisconsin, USA; 11 Department of Pathology and Laboratory Medicine, University of Connecticut Health Center, Farmington, Connecticut, USA; 12 Department of Pathology, Eastern Connecticut Health Network 5303 , Manchester, Connecticut, USA; 13 American Health Associates Laboratory, Cincinnati, Ohio, USA; 14 Cape Regional Health System 98147 , Cape May Court House, New Jersey, USA; 15 Department of Pathology and Laboratory Medicine, Weill Cornell Medicine 207092 , New York, New York, USA; 16 Division of Infectious Diseases, Hartford Hospital 23893, Hartford, Connecticut, USA; University of Fribourg, Fribourg, Switzerland

**Keywords:** Enterobacterales, outpatient centers, ceftibuten-avibactam

## Abstract

Ceftibuten is being developed in combination with the oral prodrug of avibactam. Outpatient isolates (*n* = 500) from 11 US states underwent susceptibility testing with manual broth microdilution. Ceftibuten had susceptibility rates of 64% (CLSI) and 23% (EUCAST). Ceftibuten-avibactam achieved 96.4% inhibition at MIC ≤ 1 µg/mL. Susceptibility rates were as follows: cefpodoxime (2.6%), ceftriaxone (1%), ceftazidime-avibactam (99.6%), tebipenem (89%), ertapenem (94.4%), levofloxacin (26.6%), trimethoprim-sulfamethoxazole (40.2%), and fosfomycin (96.8%). Ceftibuten-avibactam demonstrates potent *in vitro* activity against cephalosporin non-susceptible isolates.

## INTRODUCTION

Avibactam is a non-β-lactam inhibitor that targets Ambler class A β-lactamases, including ESBL, KPC, class C (AmpC), and some class D (OXA-48) β-lactamases (1, 2). An oral pro-drug formulation of avibactam has been partnered with ceftibuten, an oral third-generation cephalosporin (3GC) to treat complicated urinary tract infections (cUTI) (3). Preclinical surveillance studies are important to help define an antimicrobial’s *in vitro* activity and spectrum (4, 5). There is limited data on ceftibuten-avibactam’s *in vitro* activity outside of hospitalized patients (2, 6). The aim of this study was to assess the *in vitro* activity of ceftibuten-avibactam and eight comparator antimicrobials against clinical isolates collected from outpatients.

The study included microbiology laboratories from 12 medical centers across 11 US states. These sites collected 3GC non-susceptible isolates from outpatients between 2022 and 2024 and shipped them to the Center for Anti-Infective Research and Development (CAIRD) (Hartford, CT) for further testing. Within CAIRD, isolates were subcultured onto trypticase soy agar with 5% sheep blood plates (Becton Dickinson, Sparks, MD) and stored in skimmed milk at −80°C until susceptibility testing was conducted. Manual broth microdilution (BMD) MIC testing was performed for cefpodoxime (0.063 to 64 µg/mL), ceftriaxone (0.016 to 16 µg/mL), ceftazidime-avibactam (0.063 to 64 µg/mL), tebipenem (0.004 to 4 µg/mL), ertapenem (0.002 to 2 µg/mL), levofloxacin (0.031 to 32 µg/mL), trimethoprim-sulfamethoxazole (0.016/0.3 to 16/304 µg/mL), ceftibuten (0.063 to 64 µg/mL), and ceftibuten-avibactam (0.031 to 32 µg/mL) a single time in accordance with CLSI guidelines (7). Cation-adjusted Mueller–Hinton broth was used for the BMD studies. Disc diffusion was performed with a 200 µg fosfomycin disc containing 50 µg of glucose-6-phosphate (Sensi-Disc; Becton Dickinson, Sparks, MD) on *E. coli* isolates from the urinary tract (8). Isolates with ceftibuten-avibactam MIC >1 µg/mL underwent genotypic carbapenemase testing on the Xpert Carba-R assay (GXCARBAR-10; Cepheid, Sunnyvale, CA), as previously described (9, 10). Ceftibuten-avibactam provisional breakpoints were applied (susceptible ≤1 µg/ml; resistant >1 µg/mL). Provisional tebipenem breakpoints were applied (susceptible ≤0.125 µg/mL; intermediate 0.25 µg/mL; resistant ≥0.5 µg/mL) (11). Ceftibuten MICs were interpreted using CLSI (susceptible ≤8 µg/mL; intermediate 16 µg/mL; resistant ≥32 µg/mL) and EUCAST (susceptible ≤1 µg/mL; resistant >1 µg/mL) breakpoints, and MICs for all other comparator agents were interpreted using current CLSI breakpoints (7, 12). Isolates were characterized as 3GC non-susceptible if the MIC value to cefpodoxime was ≥2 µg/mL for *P. mirabilis* and ceftriaxone MIC was ≥2 µg/mL for all other isolates (7).

A total of 578 isolates were received from contributing sites, and 500 were confirmed in-house to meet 3GC non-susceptibility study inclusion criteria. The average patient age was 68 years, and 64% of patients were female. Forty percent of isolates were obtained from nursing homes/long-term care facilities, 17% from urology clinics, and 15% from ambulatory/primary care settings, and organisms were predominantly (90%) isolated from urine cultures (Table S1). Percent susceptibility and MIC_50/90_ for ceftibuten-avibactam and comparators against all isolates, species groups, and outpatient facilities are summarized in Tables 1 to 3. With the addition of avibactam to ceftibuten, there was a marked reduction in MIC values of ceftibuten (MIC_90_: 128 mg/mL) compared to ceftibuten-avibactam (MIC_90_: 0.125 mg/mL) (Fig. S1). Ceftibuten-avibactam and ceftazidime-avibactam were the most active agents, displaying >95% susceptibility rates (Table 2); however, the MIC_50/90_ for ceftibuten-avibactam (0.031/0.125 µg/mL) was twofold lower than that of ceftazidime-avibactam (0.25/0.5 µg/mL).

**TABLE 1:** Summary of ceftibuten-avibactam *in vitro* activity against isolates from outpatient healthcare facilities (United States, 2022–2024)

	Cumulative % of isolates inhibited at ceftibuten-avibactam MIC (µg/mL) of												MIC_50_	MIC_90_
Category (no. of isolates)	≤0.03	0.06	0.12	0.25	0.5	1	2	4	8	16	32	>32		
Study cohort (500)	62	84.2	91	93.6	95.2	96.4	97.4	98.6	99.2	99.6	99.6	100	0.03	0.12
*E. coli* (343)	59.8	87.2	95.6	98	99.1	99.7	100	100	100	100	100	100	0.03	0.12
*K. pneumoniae* (79)	72.2	88.6	93.7	96.2	98.7	98.7	98.7	98.7	98.7	98.7	98.7	100	0.03	0.12
*P. mirabilis* (37)	94.6	97.3	97.3	97.3	97.3	97.3	97.3	97.3	97.3	97.3	97.3	100	0.03	0.03

**TABLE 2:** *In vitro* activities of ceftibuten-avibactam and comparator antimicrobial agents against all isolates as well as stratified by species^*a*^

Category (no. of isolates)	Antimicrobial agent	%S	%I	%R	MIC_50_ (µg/mL)	MIC_90_ (µg/mL)
All isolates (500)	Ceftibuten-avibactam	96.4	NA^*c*^	3.6	0.03	0.12
	Ceftibuten_CLSI_	64	12.8	23.2	4	128
	Ceftibuten_EUCAST_	22.8	NA	77.2	4	128
	Cefpodoxime	2.6	1.4	96	128	128
	Ceftriaxone	1	2	97	32	32
	Ceftazidime-avibactam	99.6	NA	0.4	0.25	0.5
	Tebipenem^*b*^	89	7.6	3.4	0.02	0.25
	Ertapenem	94.4	2.4	3.2	0.03	0.5
	Levofloxacin	26.6	5.8	67.6	16	32
	Trimethoprim-sulfamethoxazole	40.2	NA	59.8	32	32
*E. coli* (343)	Ceftibuten-avibactam	99.7	NA	0.3	0.03	0.12
	Ceftibuten_CLSI_	63.9	14	22.1	4	64
	Ceftibuten_EUCAST_	20.1	NA	79.9	4	64
	Cefpodoxime	2.3	0.9	96.8	128	128
	Ceftriaxone	0.0	0.6	99.4	32	32
	Ceftazidime-avibactam	100	NA	0.0	0.25	0.5
	Tebipenem^*b*^	98	2	0.0	0.02	0.03
	Ertapenem	98.2	1.2	0.6	0.03	0.12
	Levofloxacin	18.1	2.6	79.3	16	32
	Trimethoprim-sulfamethoxazole	42.3	NA	57.7	32	32
	Fosfomycin	96.8	0.96	2.24	NA	NA
*K. pneumoniae* (79)	Ceftibuten-avibactam	98.7	NA	1.3	0.03	0.12
	Ceftibuten_CLSI_	65.8	17.7	16.5	4	64
	Ceftibuten_EUCAST_	12.7	NA	87.3	4	64
	Cefpodoxime	0.0	1.3	98.7	128	128
	Ceftriaxone	0.0	2.5	97.5	32	32
	Ceftazidime-avibactam	98.7	NA	1.3	0.5	1
	Tebipenem^*b*^	88.6	6.3	5.1	0.03	0.25
	Ertapenem	93.6	1.3	5.1	0.06	0.5
	Levofloxacin	38	19	43	1	16
	Trimethoprim-sulfamethoxazole	20.3	NA	79.7	32	32
*P. mirabilis* (37)	Ceftibuten-avibactam	97.3	NA	2.7	0.03	0.03
	Ceftibuten_CLSI_	94.6	2.7	2.7	0.12	8
	Ceftibuten_EUCAST_	70.3	NA	29.7	0.12	8
	Cefpodoxime	8.1	2.7	89.2	128	128
	Ceftriaxone	13.5	5.4	81.1	32	32
	Ceftazidime-avibactam	97.3	NA	2.7	0.06	0.25
	Tebipenem^*b*^	29.7	48.7	21.6	0.25	0.5
	Ertapenem	91.9	2.7	5.4	0.03	0.5
	Levofloxacin	18.9	5.4	75.7	8	32
	Trimethoprim-sulfamethoxazole	21.6	NA	78.4	32	32

^*a*^ Ceftibuten assessed with both CLSI and EUCAST breakpoints

^*b*^ Preliminary susceptible breakpoint ≤0.125 µg/mL.

^*c*^ NA, not applicable.

**TABLE 3:** *In vitro* activities of ceftibuten-avibactam and comparator antimicrobial agents stratified by outpatient location^*a*^

Outpatient healthcare facility (no. of isolates)	Antimicrobial agent	%S	%I	%R	MIC_50_ (µg/mL)	MIC_90_ (µg/mL)
Nursing home/long-term care facility (198)	Ceftibuten-avibactam	96.5	NA^*d*^	3.5	0.03	0.06
	Ceftibuten_CLSI_	71	10.5	18.5	4	64
	Ceftibuten_EUCAST_	26.3	NA	73.7	4	64
	Cefpodoxime	1	1.5	97.5	128	128
	Ceftriaxone	1.5	2	96.5	32	32
	Ceftazidime-avibactam	99	NA	1	0.25	0.5
	Tebipenem^*b*^	86	9	5	0.03	0.25
	Ertapenem	93	3	4	0.06	0.5
	Levofloxacin	18	6.5	75.5	16	32
	Trimethoprim-sulfamethoxazole	33	NA	67	32	32
	Fosfomycin^*c*^	95	2.5	2.5	NA	NA
Urology clinic (87)	Ceftibuten-avibactam	98	NA	2	0.03	0.25
	Ceftibuten_CLSI_	61	14	25	4	128
	Ceftibuten_EUCAST_	16.1	NA	83.9	4	128
	Cefpodoxime	0	1	99	128	128
	Ceftriaxone	0	1	99	32	32
	Ceftazidime-avibactam	100	NA	0	0.03	0.25
	Tebipenem^*b*^	91	8	1	0.03	0.12
	Ertapenem	95.4	0	4.6	0.06	0.5
	Levofloxacin	32	8	60	8	32
	Trimethoprim-sulfamethoxazole	39	NA	61	32	32
	Fosfomycin^*c*^	96.8	1.6	1.6	NA	NA
Ambulatory/primary care clinic (77)	Ceftibuten-avibactam	95	NA	5	0.03	0.25
	Ceftibuten_CLSI_	57	14	29	8	128
	Ceftibuten_EUCAST_	26	NA	74	8	128
	Cefpodoxime	9	1	90	128	128
	Ceftriaxone	1	3	96	32	32
	Ceftazidime-avibactam	100	NA	0	0.25	0.5
	Tebipenem^*b*^	93.5	6.5	0	0.02	0.12
	Ertapenem	95	34	1	0.03	0.5
	Levofloxacin	28.6	5.2	66.2	16	32
	Trimethoprim-sulfamethoxazole	56	NA	44	0.5	32
	Fosfomycin^*c*^	98.4	0	1.6	NA	NA
Emergency department/urgent care (36)	Ceftibuten-avibactam	94.4	NA	5.6	0.03	0.5
	Ceftibuten_CLSI_	61	8	31	8	128
	Ceftibuten_EUCAST_	19.4	NA	80.6	8	128
	Cefpodoxime	0	3	97	128	128
	Ceftriaxone	2.8	2.8	94.4	32	32
	Ceftazidime-avibactam	100	NA	0	0.25	1
	Tebipenem^*b*^	83.3	11.1	5.6	0.02	0.25
	Ertapenem	94.4	5.6	0	0.03	0.5
	Levofloxacin	38.9	2.8	58.3	8	32
	Trimethoprim-sulfamethoxazole	47.2	NA	52.8	16	32
	Fosfomycin^*c*^	100	0	0	NA	NA

^*a*^ Ceftibuten assessed with both CLSI and EUCAST breakpoints

^*b*^ Preliminary susceptible breakpoint ≤0.125 µg/mL.

^*c*^ For *E. coli* isolates from urine source only using disc diffusion.

^*d*^ NA, not applicable.

Susceptibility rates for ceftibuten, cefpodoxime, and ceftriaxone were low (1% to 64%). Tebipenem and ertapenem demonstrated high *in vitro* activity, inhibiting 89% and 94% of isolates, respectively. Levofloxacin and trimethoprim-sulfamethoxazole demonstrated *in vitro* activity rates of 27% and 40%, respectively. Fosfomycin exhibited a susceptibility rate of 97% against *E. coli* isolates from urinary tract infections. Across the three predominant species (*E. coli*, *K. pneumoniae*, and *P. mirabilis*), high susceptibility rates (97% to 100%) to ceftibuten-avibactam were observed at ≤1 µg/mL. Ceftibuten-avibactam also demonstrated high susceptibility rates (94% to 98%) against organisms irrespective of outpatient location (Table 3). The percent susceptibility and MIC_50/90_ for ceftibuten-avibactam and comparator agents against less frequently encountered species are listed in Tables S2 and S3. Among the 500 isolates tested, 18 (3.6%) exhibited non-susceptibility (MIC >1 µg/mL) to ceftibuten-avibactam (Table S4). The Carba-R test identified the presence of NDM in one isolate.

Ceftibuten-avibactam could serve as a carbapenem-sparing option and enable more prompt transitions of care by taking advantage of this novel parenteral to oral β-lactam/β-lactamase inhibitor switch option (13, 14). With the rise of ESBL infections, effective outpatient options are limited (15–19), which has led to increased hospitalizations (20–24). Moreover, the available oral agents, such as levofloxacin and trimethoprim-sulfamethoxazole, demonstrate limited activity against ESBL-phenotype isolates, while oral fosfomycin is limited to urine-based *E. coli,* thus lacking a broad spectrum profile (2).

In the current study, ceftibuten-avibactam inhibited >95% of isolates resulting in MIC_50/90_ values against 3GC non-susceptible isolates compared to the carbapenems tested. Ceftibuten-avibactam *in vitro* activity was consistent across various patient locations, inhibiting 94.4% to 98% of isolates in four major outpatient settings: nursing homes and long-term care facilities, urology clinics, emergency and urgent care centers, and women’s health clinics. Ceftibuten-avibactam exhibited potent inhibitory effects against *E. coli* (99.7%) and *K. pneumoniae* (98.7%) which represent the predominant Gram-negative species causing infections (25–27).

It is worth noting that ceftibuten does not have harmonized CLSI and EUCAST MIC breakpoints. Per EUCAST breakpoints, only 23% of isolates were susceptible to ceftibuten in the current study. However, using CLSI breakpoints, 64% of isolates were susceptible. The carbapenemase-focused genotypic testing performed on the subset of isolates with ceftibuten-avibactam MIC ≥1 µg/mL highlighted the rare presence of *bla*_NDM_ (*n* = 1). Future studies ascertaining other contributing resistance mechanisms, such as AmpC hyperproduction and/or a combination of alterations to PBP3 and uptake/efflux components, are warranted (28, 29). In the current study, most ceftibuten-avibactam non-susceptible isolates were *Enterobacter* species (*n* = 14), known for AmpC overproduction (30). Furthermore, the assessment of additional comparator agents, such as amikacin and nitrofurantoin, against a similar challenge set of organisms is needed.

We conclude that ceftibuten-avibactam was active against a large collection of contemporary 3GC non-susceptible isolates from patients in diverse outpatient settings, including nursing homes.

## SUPPLEMENTAL MATERIAL

Supplemental material aac.01867-24-s0001.docx Tables S1 to S4; Fig. S1.

ASM does not own the copyrights to Supplemental Material that may be linked to, or accessed through, an article. The authors have granted ASM a non-exclusive, world-wide license to publish the Supplemental Material files. Please contact the corresponding author directly for reuse.
