# Supplementary material for: Activity of novel ceftibuten-avibactam, ceftazidime-avibactam, and comparators against a challenge set of Enterobacterales from outpatient centers and nursing homes across the United States (2022–2024)
Source: Antimicrob Agents Chemother. 2025 Apr 3;69(5):e01867-24. doi: 10.1128/aac.01867-24 (PMC12057334; doi:10.1128/aac.01867-24)

Supplemental Data – March 24, 2025

**Activity of Novel Ceftibuten-Avibactam, Ceftazidime-Avibactam, and Comparators against a challenge set of Enterobacterales from Outpatient Centers and Nursing Homes across the United States (2022-2024)**

**RESULTS**

**Table S1.** Demographic data for the patients corresponding to submitted isolates (n = 500)

| **Characteristics** | **Mean (SD) or n (%)** |
| --- | --- |
| **Age (Years), mean (SD)** | 68 (18) |
| **Female, n (%)** | 321 (64%) |
| **Outpatient location, n (%)** |  |
| Nursing Home/Long-Term Care Facility | 198 (40%) |
| Urology clinic | 87 (17%) |
| Ambulatory/Primary care clinic | 77 (15%) |
| Emergency Department/Urgent Care Center | 36 (7%) |
| Women's Health clinic | 13 (3%) |
| Other clinics^a^ | 89 (18%) |
| **Culture Source** |  |
| Urine | 450 (90%) |
| Wound/Soft Tissue | 26 (5%) |
| Blood | 15 (3%) |
| Other source^b^ | 9 (2%) |

^a^ Other clinics: Rheumatology, Geriatrics, Neurology, Cardiology

^b^ other source: Genital, Respiratory, Ear-drainage, Sputum

**Table S2.** Summary of Ceftibuten-avibactam *in vitro* activity against less frequently encountered isolates from outpatient healthcare facilities (United States, 2022-2024)

| Category (no. of isolates) | Cumulative % of isolates inhibited at ceftibuten-avibactam MIC (µg/mL) of | | | | | | | | | | | | MIC_50_ | MIC_90_ |
| --- | --- | --- | --- | --- | --- | --- | --- | --- | --- | --- | --- | --- | --- | --- |
|  | ≤ 0.03 | 0.06 | 0.12 | 0.25 | 0.5 | 1 | 2 | 4 | 8 | 16 | 32 | >32 |  |  |
| *Enterobacter cloacae* (17) | 0.0 | 5.9 | 5.9 | 5.9 | 5.9 | 23.5 | 41.2 | 70.6 | 88.2 | 100 | 100 | 100 | 4 | 16 |
| *Citrobacter freundii* (7) | 42.9 | 42.9 | 42.9 | 85.7 | 100 | 100 | 100 | 100 | 100 | 100 | 100 | 100 | 0.25 | 0.5 |
| *Klebsiella aerogenes* (6) | 0.0 | 16.7 | 33.3 | 33.3 | 50 | 66.7 | 100 | 100 | 100 | 100 | 100 | 100 | 0.5 | 2 |
| *Klebsiella oxytoca* (5) | 80 | 100 | 100 | 100 | 100 | 100 | 100 | 100 | 100 | 100 | 100 | 100 | 0.03 | 0.06 |
| *Proteus vulgaris* (2) | 100 | 100 | 100 | 100 | 100 | 100 | 100 | 100 | 100 | 100 | 100 | 100 | 0.03 | 0.03 |
| *Citrobacter koseri* (1) | 100 | 100 | 100 | 100 | 100 | 100 | 100 | 100 | 100 | 100 | 100 | 100 | 0.03 | 0.03 |
| *Providencia stuartii* (1) | 100 | 100 | 100 | 100 | 100 | 100 | 100 | 100 | 100 | 100 | 100 | 100 | 0.03 | 0.03 |
| *Proteus penneri* (1) | 100 | 100 | 100 | 100 | 100 | 100 | 100 | 100 | 100 | 100 | 100 | 100 | 0.03 | 0.03 |
| *Serratia marcescens* (1) | 100 | 100 | 100 | 100 | 100 | 100 | 100 | 100 | 100 | 100 | 100 | 100 | 0.03 | 0.03 |

**Table S3.** *In vitro* activities of ceftibuten-avibactam and comparator antimicrobial agents against all isolates stratified by species

| **Category (no. of isolates)** | **Antimicrobial agent** | **%S** | **%I** | **%R** | **MIC_50_ (µg/mL)** | **MIC_90_ (µg/mL)** |
| --- | --- | --- | --- | --- | --- | --- |
| ***Enterobacter cloacae* (17)** | Ceftibuten-avibactam | 23.5 | NA | 76.5 | 4 | 16 |
|  | Ceftibuten_CLSI_ | 11.8 | 0.0 | 88.2 | 128 | 128 |
|  | Ceftibuten_EUCAST_ | 5.9 | NA | 94.1 | 128 | 128 |
|  | Cefpodoxime | 0.0 | 0.0 | 100 | 128 | 128 |
|  | Ceftriaxone | 0.0 | 5.9 | 94.1 | 32 | 32 |
|  | Ceftazidime-avibactam | 100 | NA | 0.0 | 1 | 2 |
|  | Tebipenem^a^ | 58.8 | 23.5 | 17.7 | 0.12 | 0.5 |
|  | Ertapenem | 35.3 | 29.4 | 35.3 | 1 | 2 |
|  | Levofloxacin | 100 | 0.0 | 0.0 | 0.06 | 0.06 |
|  | Trimethoprim-sulfamethoxazole | 94.1 | NA | 5.9 | 0.12 | 0.5 |
| ***Citrobacter freundii* (7)** | Ceftibuten-avibactam | 100 | NA | 0.0 | 0.25 | 0.5 |
|  | Ceftibuten_CLSI_ | 28.6 | 0.0 | 71.4 | 128 | 128 |
|  | Ceftibuten_EUCAST_ | 0.0 | NA | 100 | 128 | 128 |
|  | Cefpodoxime | 0.0 | 28.6 | 71.4 | 128 | 128 |
|  | Ceftriaxone | 0.0 | 28.6 | 71.4 | 32 | 32 |
|  | Ceftazidime-avibactam | 100 | NA | 0.0 | 0.25 | 1 |
|  | Tebipenem^a^ | 100 | 0.0 | 0.0 | 0.06 | 0.06 |
|  | Ertapenem | 100 | 0.0 | 0.0 | 0.12 | 0.25 |
|  | Levofloxacin | 17.4 | 14.3 | 14.3 | 0.25 | 1 |
|  | Trimethoprim-sulfamethoxazole | 71.4 | NA | 28.6 | 0.12 | 32 |
| ***Klebsiella aerogenes* (6)** | Ceftibuten-avibactam | 66.7 | NA | 33.3 | 0.5 | 2 |
|  | Ceftibuten_CLSI_ | 16.7 | 0.0 | 83.3 | 128 | 128 |
|  | Ceftibuten_EUCAST_ | 16.7 | 0.0 | 83.3 | 128 | 128 |
|  | Cefpodoxime | 0.0 | 0.0 | 100 | 128 | 128 |
|  | Ceftriaxone | 0.0 | 16.7 | 83.3 | 32 | 32 |
|  | Ceftazidime-avibactam | 100 | NA | 0.0 | 0.5 | 2 |
|  | Tebipenem^a^ | 50 | 33.3 | 16.7 | 0.12 | 0.5 |
|  | Ertapenem | 50 | 16.7 | 33.3 | 0.5 | 2 |
|  | Levofloxacin | 83.3 | 0.0 | 16.7 | 0.12 | 64 |
|  | Trimethoprim-sulfamethoxazole | 83.3 | 0.0 | 16.7 | 0.12 | 32 |
| ***Klebsiella oxytoca* (5)** | Ceftibuten-avibactam | 100 | NA | 0.0 | 0.03 | 0.63 |
|  | Ceftibuten_CLSI_ | 60 | 20 | 20 | 8 | 32 |
|  | Ceftibuten_EUCAST_ | 20 | NA | 80 | 8 | 32 |
|  | Cefpodoxime | 0.0 | 0.0 | 100 | 128 | 128 |
|  | Ceftriaxone | 0.0 | 0.0 | 100 | 32 | 32 |
|  | Ceftazidime-avibactam | 100 | NA | 0.0 | 1 | 2 |
|  | Tebipenem^a^ | 100 | 0.0 | 0.0 | 0.03 | 0.63 |
|  | Ertapenem | 100 | 0.0 | 0.0 | 0.12 | 0.5 |
|  | Levofloxacin | 60 | 40 | 0.0 | 0.5 | 1 |
|  | Trimethoprim-sulfamethoxazole | 20 | NA | 80 | 32 | 32 |
| ***Proteus vulgaris* (2)** | Ceftibuten-avibactam | 100 | NA | 0.0 | 0.03 | 0.03 |
|  | Ceftibuten_CLSI_ | 100 | 0.0 | 0.0 | 0.06 | 0.06 |
|  | Ceftibuten_EUCAST_ | 100 | NA | 0.0 | 0.06 | 0.06 |
|  | Cefpodoxime | 50 | 0.0 | 50 | 0.5 | 16 |
|  | Ceftriaxone | 00.0 | 0.0 | 100 | 32 | 32 |
|  | Ceftazidime-avibactam | 100 | NA | 0.0 | 0.06 | 0.06 |
|  | Tebipenem^a^ | 0.0 | 100 | 0.0 | 0.25 | 0.25 |
|  | Ertapenem | 100 | 0.0 | 0.0 | 0.25 | 0.25 |
|  | Levofloxacin | 100 | 0.0 | 0.0 | 0.03 | 0.06 |
|  | Trimethoprim-sulfamethoxazole | 100 | NA | 0.0 | 0.12 | 0.12 |
| ***Citrobacter koseri* (1)** | Ceftibuten-avibactam | 100 | NA | 0.0 | 0.03 | 0.03 |
|  | Ceftibuten_CLSI_ | 100 | 0.0 | 0.0 | 0.06 | 0.06 |
|  | Ceftibuten_EUCAST_ | 100 | NA | 0.0 | 0.06 | 0.06 |
|  | Cefpodoxime | 0.0 | 0.0 | 100 | 128 | 128 |
|  | Ceftriaxone | 0.0 | 0.0 | 100 | 16 | 16 |
|  | Ceftazidime-avibactam | 100 | NA | 0.0 | 0.12 | 0.12 |
|  | Tebipenem^a^ | 100 | 0.0 | 0.0 | 0.02 | 0.02 |
|  | Ertapenem | 100 | 0.0 | 0.0 | 0.25 | 0.25 |
|  | Levofloxacin | 0.0 | 0.0 | 100 | 2 | 2 |
|  | Trimethoprim-sulfamethoxazole | 100 | NA | 0.0 | 0.12 | 0.12 |
| ***Providencia stuartii* (1)** | Ceftibuten-avibactam | 100 | NA | 0.0 | 0.03 | 0.03 |
|  | Ceftibuten_CLSI_ | 100 | 0.0 | 0.0 | 0.06 | 0.06 |
|  | Ceftibuten_EUCAST_ | 100 | NA | 0.0 | 0.06 | 0.06 |
|  | Cefpodoxime | 100 | 0.0 | 0.0 | 1 | 1 |
|  | Ceftriaxone | 0.0 | 0.0 | 100 | 4 | 4 |
|  | Ceftazidime-avibactam | 100 | NA | 0.0 | 0.25 | 0.25 |
|  | Tebipenem^a^ | 0.0 | 0.0 | 100 | 1 | 1 |
|  | Ertapenem | 100 | 0.0 | 0.0 | 0.5 | 0.5 |
|  | Levofloxacin | 100 | 0.0 | 0.0 | 0.12 | 0.12 |
|  | Trimethoprim-sulfamethoxazole | 100 | NA | 0.0 | 0.06 | 0.06 |
| ***Proteus penneri* (1)** | Ceftibuten-avibactam | 100 | NA | 0.0 | 0.03 | 0.03 |
|  | Ceftibuten_CLSI_ | 100 | 0.0 | 0.0 | 0.06 | 0.06 |
|  | Ceftibuten_EUCAST_ | 100 | NA | 0.0 | 0.06 | 0.06 |
|  | Cefpodoxime | 0.0 | 0.0 | 100 | 64 | 64 |
|  | Ceftriaxone | 0.0 | 0.0 | 100 | 32 | 32 |
|  | Ceftazidime-avibactam | 100 | NA | 0.0 | 0.06 | 0.06 |
|  | Tebipenem^a^ | 100 | 0.0 | 0.0 | 0.12 | 0.12 |
|  | Ertapenem | 100 | 0.0 | 0.0 | 0.03 | 0.03 |
|  | Levofloxacin | 100 | 0.0 | 0.0 | 0.03 | 0.03 |
|  | Trimethoprim-sulfamethoxazole | 100 | NA | 0.0 | 0.25 | 0.25 |
| ***Serratia marcescens* (1)** | Ceftibuten-avibactam | 100 | NA | 0.0 | 0.03 | 0.03 |
|  | Ceftibuten_CLSI_ | 100 | 0.0 | 0.0 | 0.12 | 0.12 |
|  | Ceftibuten_EUCAST_ | 100 | NA | 0.0 | 0.12 | 0.12 |
|  | Cefpodoxime | 0.0 | 0.0 | 100 | 128 | 128 |
|  | Ceftriaxone | 0.0 | 0.0 | 100 | 32 | 32 |
|  | Ceftazidime-avibactam | 100 | NA | 0.0 | 0.25 | 0.25 |
|  | Tebipenem^a^ | 100 | 0.0 | 0.0 | 0.02 | 0.02 |
|  | Ertapenem | 100 | 0.0 | 0.0 | 0.06 | 0.06 |
|  | Levofloxacin | 0.0 | 0.0 | 100 | 16 | 16 |
|  | Trimethoprim-sulfamethoxazole | 0.0 | NA | 100 | 32 | 32 |

Ceftibuten assessed with both CLSI and EUCAST breakpoints

^a^ Preliminary susceptible breakpoint ≤0.125 µg/mL

NA, not applicable

**Table S4.** MIC values, species and carba-R results for the 18 ceftibuten-avibactam- non susceptible isolates (MIC >1 µg/mL).

| **ID** | **Species** | **Carba-R Results** | **MIC µg/mL** | | | | | | | | |
| --- | --- | --- | --- | --- | --- | --- | --- | --- | --- | --- | --- |
|  |  |  | **CTB** | **CTB-AVI** | **CAZ-AVI** | **LVX** | **CPD** | **CRO** | **ETP** | **TBP** | **TMP-SMX** |
| 1 | ***Enterobacter cloacae*** | IMP, VIM, NDM, KPC, OXA 48 - Not detected | >64 | 2 | 1 | 0.03 | >64 | >16 | 1 | 0.06 | 0.12 |
| 2 | ***Klebsiella pneumoniae*** | IMP, VIM, KPC, OXA 48 - Not detected; **NDM Detected** | >64 | >32 | >64 | 1 | >64 | >16 | >2 | >4 | >16 |
| 3 | ***Enterobacter cloacae*** | IMP, VIM, NDM, KPC, OXA 48 - Not detected | >64 | 4 | 2 | 0.06 | >64 | >16 | 1 | 0.12 | 0.12 |
| 4 | ***Klebsiella aerogenes*** | IMP, VIM, NDM, KPC, OXA 48 - Not detected | >64 | 2 | 2 | 0.12 | >64 | >16 | 2 | 0.25 | 0.12 |
| 5 | ***Klebsiella aerogenes*** | IMP, VIM, NDM, KPC, OXA 48 - Not detected | >64 | 2 | 2 | 0.25 | >64 | >16 | 2 | 0.25 | 0.03 |
| 6 | ***Enterobacter cloacae*** | IMP, VIM, NDM, KPC, OXA 48 - Not detected | 8 | 4 | 8 | 0.06 | 64 | 8 | 0.5 | 0.03 | 0.03 |
| 7 | ***Enterobacter cloacae*** | IMP, VIM, NDM, KPC, OXA 48 - Not detected | >64 | 8 | 1 | 0.06 | >64 | >16 | 2 | 0.25 | 0.12 |
| 8 | ***Enterobacter cloacae*** | IMP, VIM, NDM, KPC, OXA 48 - Not detected | >64 | 16 | 2 | 0.06 | >64 | >16 | >2 | 0.5 | 0.12 |
| 9 | ***Enterobacter cloacae*** | IMP, VIM, NDM, KPC, OXA 48 - Not detected | >64 | 8 | 2 | 0.06 | >64 | >16 | 2 | 0.12 | 0.12 |
| 10 | ***Enterobacter cloacae*** | IMP, VIM, NDM, KPC, OXA 48 - Not detected | >64 | 8 | 2 | 0.06 | >64 | >16 | 2 | 0.5 | >16 |
| 11 | ***Escherichia coli*** | IMP, VIM, NDM, KPC, OXA 48 - Not detected | >64 | 4 | 2 | 16 | >64 | >16 | 0.06 | 0.01 | >16 |
| 12 | ***Enterobacter cloacae*** | IMP, VIM, NDM, KPC, OXA 48 - Not detected | >64 | 4 | 1 | 0.06 | >64 | >16 | 1 | 0.25 | 0.5 |
| 13 | ***Enterobacter cloacae*** | IMP, VIM, NDM, KPC, OXA 48 - Not detected | >64 | 2 | 1 | 0.06 | >64 | >16 | 0.5 | 0.12 | 0.25 |
| 14 | ***Enterobacter cloacae*** | IMP, VIM, NDM, KPC, OXA 48 - Not detected | >64 | 2 | 1 | 0.06 | >64 | >16 | 0.5 | 0.06 | 0.12 |
| 15 | ***Enterobacter cloacae*** | IMP, VIM, NDM, KPC, OXA 48 - Not detected | >64 | 4 | 1 | 0.06 | >64 | >16 | 0.5 | 0.06 | 0.12 |
| 16 | ***Enterobacter* cloacae** | IMP, VIM, NDM, KPC, OXA 48 - Not detected | >64 | 16 | 2 | 0.06 | >64 | >16 | 2 | 1 | 0.06 |
| 17 | ***Enterobacter cloacae*** | IMP, VIM, NDM, KPC, OXA 48 - Not detected | >64 | 4 | 0.5 | 0.03 | >64 | >16 | 1 | 0.25 | 0.06 |
| 18 | ***Proteus mirabilis*** | IMP, VIM, NDM, KPC, OXA 48 - Not detected | >64 | >32 | >64 | >32 | >64 | >16 | >2 | 1 | >16 |

CTB: ceftibuten; AVI: avibactam; CAZ: ceftazidime; LVX: levofloxacin; CPD: cefpodoxime; CRO: ceftriaxone; ETP: ertapenem; TBP: tebipenem; TMP-SMX: trimethoprim-sulfamethoxazole.

**Figure S1.** MIC distributions for 500 3^rd^-generation cephalosporin non-susceptible isolates


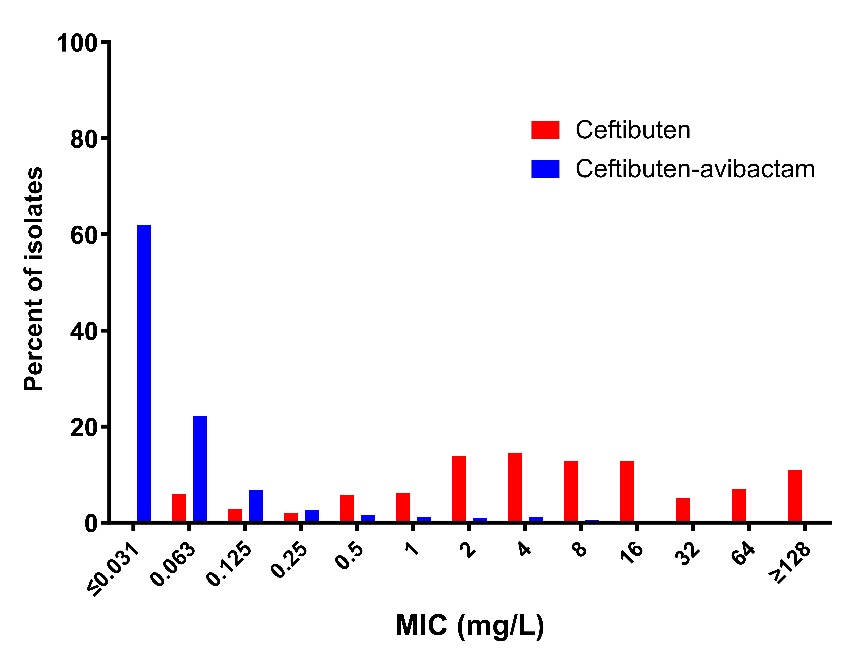

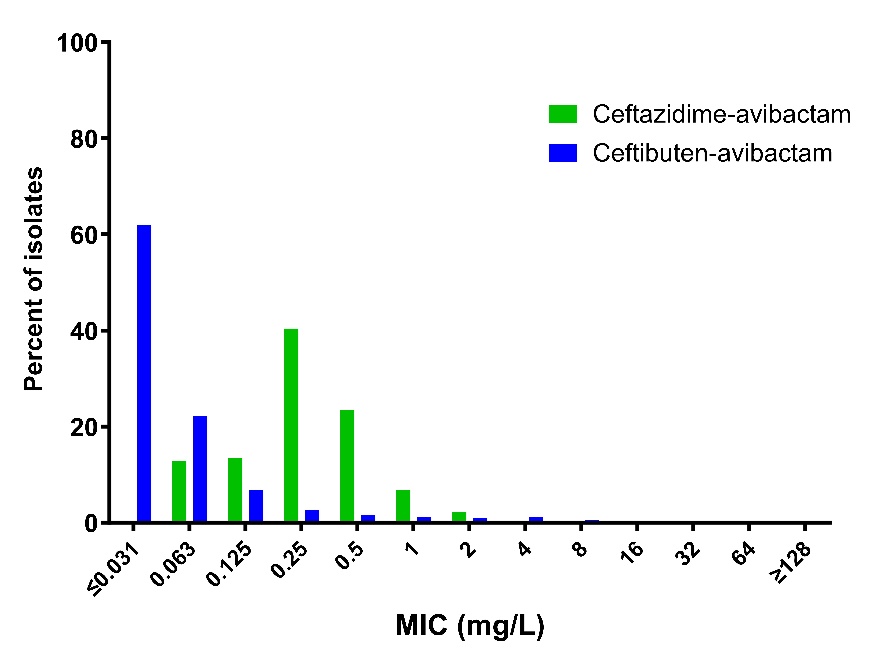

Supplement: Supplemental material — Tables S1 to S4; Fig. S1. [file aac.01867-24-s0001.docx]
